# Quality assessment of expert answers to lay questions about cystic fibrosis from various language zones in Europe: the ECORN-CF project

**DOI:** 10.1186/1471-2288-12-11

**Published:** 2012-02-06

**Authors:** Daniela d'Alquen, Kris De Boeck, Judy Bradley, Věra Vávrová, Birgit Dembski, Thomas OF Wagner, Annette Pfalz, Helge Hebestreit

**Affiliations:** 1 Department of Pediatrics, University Hospital Wuerzburg, Josef-Schneider-Str. 2, 97080 Wuerzburg, Germany; 2 Pediatric Pulmonology, Department of Pediatrics, University Hospital of Leuven, Herestraat 49, 3000 Leuven, Belgium; 3 Health and Rehabilitation Sciences Research Institute, University of Ulster and Adult CF Centre, Belfast Health and Social Care Trust, Shore Road, Newtownabbey BT37 0QB, Northern Ireland; 4 Cystic Fibrosis Centre, Charles University, 2nd School of Medicine, V Uvalu 84, 15006 Prague 5, Czech Republic; 5 CF Europe, Bonn, and CF Association, Mukoviszidose e.V., In den Dauen 6, 53117 Bonn, Germany; 6 Department of Pneumology, Goethe University Hospital Frankfurt, Theodor-Stern-Kai 7, 60590 Frankfurt am Main, Germany

## Abstract

**Background:**

The European Centres of Reference Network for Cystic Fibrosis (ECORN-CF) established an Internet forum which provides the opportunity for CF patients and other interested people to ask experts questions about CF in their mother language. The objectives of this study were to: 1) develop a detailed quality assessment tool to analyze quality of expert answers, 2) evaluate the intra- and inter-rater agreement of this tool, and 3) explore changes in the quality of expert answers over the time frame of the project.

**Methods:**

The quality assessment tool was developed by an expert panel. Five experts within the ECORN-CF project used the quality assessment tool to analyze the quality of 108 expert answers published on ECORN-CF from six language zones. 25 expert answers were scored at two time points, one year apart. Quality of answers was also assessed at an early and later period of the project. Individual rater scores and group mean scores were analyzed for each expert answer.

**Results:**

A scoring system and training manual were developed analyzing two quality categories of answers: content and formal quality. For content quality, the grades based on group mean scores for all raters showed substantial agreement between two time points, however this was not the case for the grades based on individual rater scores. For formal quality the grades based on group mean scores showed only slight agreement between two time points and there was also poor agreement between time points for the individual grades. The inter-rater agreement for content quality was fair (mean kappa value 0.232 ± 0.036, p < 0.001) while only slight agreement was observed for the grades of the formal quality (mean kappa value 0.105 ± 0.024, p < 0.001). The quality of expert answers was rated high (four language zones) or satisfactory (two language zones) and did not change over time.

**Conclusions:**

The quality assessment tool described in this study was feasible and reliable when content quality was assessed by a group of raters. Within ECORN-CF, the tool will help ensure that CF patients all over Europe have equal possibility of access to high quality expert advice on their illness.

## Background

Cystic fibrosis (CF) is an autosomal recessive inherited disease caused by mutation of the cystic fibrosis transmembrane conductance regulator (*CFTR*) gene, coding for a protein functioning as a transmembrane epithelial chloride channel [1]. It is a multi-system disease characterized by progressive pulmonary damage leading to respiratory failure, pancreatic dysfunction, liver disease, gut motility problems and elevated sweat electrolytes. CF occurs world-wide and is the most common autosomal recessive lethal hereditary disorder in Caucasians with an incidence of approximately one in 2500 live births [2,3].

A large multinational study on health which included 29025 CF patients across 35 European countries suggests that the quality of care for CF patients is not equal across Europe [4]. In some of the Eastern European countries drugs, equipment and specialized care are not always easily available. In new EU member states, specialized CF centres tend to be located in the most populated areas with access to specialized care for patients living in remote areas difficult or absent. Data available from 2003 on the median gross domestic product per person, a surrogate for health care spending, was nine times higher in EU countries than in non-EU countries [5]. The demographic data from the multinational European study reveals that CF patients from EU countries had a better survival than CF patients from non-EU countries in 2003 [4]. To overcome some of these differences between European countries, the "European Centres of Reference Network for Cystic Fibrosis" (ECORN-CF) project was initiated. The aim of ECORN-CF was to facilitate access to specialized health care counseling for all European CF patients and allow easy access to expert advice in all member state languages. The project uses the Internet as a medium for communication. This approach appears reasonable, as Internet access for patients rose significantly in the last 10 years [6,7] and the Internet has become an important medium for patients to get advice on their illness [8,9].

An Internet platform was established in the participating language zones (Czech, Dutch, English, German, Greek, Lithuanian, Polish, Romanian and Swedish; later, in October 2010, the French language zone joined the expert advice system). This Internet platform enables CF patients/lay people as well as clinicians to get expert advice/answers to questions they pose in their mother language.

The ECORN-CF patient advice platform is designed to include a two-stage central quality control mechanism. Firstly, expert answers are assessed on a national/language-zone level (national platform) and secondly on a European level (central English archive). At the national level, the quality of the expert answer is assessed by a national moderator. All expert answers are then translated into English and a European moderator/coordinator assesses the accuracy. The accuracy of the expert answers is checked (i.e. their conformance to existing guidelines) and judged as "good", "with flaws" or "unacceptable". In case an answer was not scored as "good", feedback is given to the expert, and a proposal for an amended/extended answer is provided according to published clinical guidelines. The aim of providing feedback is to improve the quality of answers over time by a training effect and provide high quality expert answers. The final approved expert answer is published on both the national platform in the mother language of the questioner, and also in English in the central archive. If the questioners provide an e-mail address, they are informed of the published answer. If no guidelines on a particular area exist, then answers are based on expert opinion. Such questions are marked in the archive to indicate the need for a consensus and development of guidelines.

A comprehensive literature review highlighted that there are currently a number of Internet based patient information/advice platforms in a range of chronic diseases [8-11], however to our knowledge there are no published studies assessing quality of expert advice. The specific objectives of this study were to: 1) develop a detailed quality assessment tool that could quantify the quality of expert answers provided within the ECORN-CF project, 2) evaluate the intra- and inter-rater agreement of the quality assessment tool, and 3) explore changes in quality of expert answers over the time frame of the ECORN-CF project.

## Methods

### 1. Development of a quality assessment tool to analyze quality of expert answers

Two main categories of study quality were included in the quality assessment tool; content quality and formal quality. The category content quality included three sub-scores to assess the correctness of the content, completeness and openness of the answer. The category formal quality included three sub-scores to assess comprehensibility, extent of the answer and the way the questioner was addressed. The above items were delineated and weighted according to the importance of the item within the category. A scale was defined to determine three grades ("good", "satisfactory" and "poor") according to the total score given. This first draft of the scoring system was presented at the first quality round table of the ECORN-CF project team on August 22^nd^, 2007. At this meeting experts reviewed the proposed scoring system and pilot tested it on a number of sample question/answers. They provided feedback on key aspects of content and formal quality and the proposed scoring system. Experts at the round table reported that the quality assessment tool had good face and content validity and was easy to use. Consensus was also reached on the content of a training manual. This training manual included guidelines on each specific aspect of quality assessment and also provided worked examples for users (see Additional File 1).

Out of all nine language zones participating in ECORN-CF only six zones could be included in this study (Czech, Dutch, English, German, Lithuanian, and Romanian). The remaining three language zones (Greek, Polish and Swedish) were excluded due to a small number of questions or a late start date of the national website.

A total of 108 expert answers to questions posed by patients or lay people from the start of the online period of the respective language zone until the 3^rd ^of July, 2009 (details see Table 1) were included in the analysis. Answers to questions posed by health care professionals were excluded. The answers sent to the central English archive by the national moderators prior to any modifications by the European coordinator were anonymized and any information that would identify the country of origin was removed to ensure that expert raters were blinded. The order of expert answers from different countries were randomly sorted and sent to the experts to score in August 2009.

**Table 1:** Selection of expert answers from participating language zones and respective time intervals

Language zone	Online since	No of answers early period (time interval)	No of answers later period (time interval)	Time span from begin of early to end of later period	Time span from end of early to begin of later period
**Czech**	October 1, 2007	10	10	18.5 months	10.5 months
		(7.11.07-29.1.08)	(12.12.08-25.5.09)		

**Dutch**	March 5, 2008	10	10	15 months	6 months
		(17.3.08-11.10.08)	(12.4.09-14.6.09)		

**English**	October 1, 2007	10	10	19.5 months	4.3 months
		(20.11.07-23.10.08)	(2.3.09-3.7.09)		

**German**	October 1, 2007	10	10	20 months	18 months
		(1.11.07-7.12.07)	(2.6.09-28.6.09)		

Lithuanian*	January 31, 2008	5	8	16.3 months	7.3 months
		(17.1.08-2.4.08)	(12.11.08-28.5.09)		

Romanian*	December 19, 2007	5	10	17.8 months	5 months
		(1.12.07-21.6.08)	(16.11.08-21.5.09)		

*Less than 10 question/answer pairs were available in some time periods.

25 out of the 108 expert answers were scored twice by each of the raters with a one year time interval. The first scoring process took place in August 2008 for the quality round table in Frankfurt in November, 2008. In August 2009 expert answers were selected as described above (including the 25 expert answers previously scored) and sent to the experts to score. The experts were directed not to look up their old records and it was assumed that they did not remember their former scores.

### 2. Assessment of intra- and inter-rater agreement

Five experts with different professional backgrounds used the newly developed scoring system and training manual to score each answer. VV (Czech Republic), KD (Belgium) and HH (Germany) represented pediatric pulmonologists specialized in CF care for children and adults and JB (UK) represented respiratory physiotherapists specialized in CF care. BD (Germany) represented the German CF-patient organization and not being a care team member scored only the formal aspects of the quality of answers.

#### 2.1 Intra-rater agreement

For each rater, the percentage of expert answers scored with the same grade on the two occasions and one or two grades lower or higher were calculated. Furthermore, an average score for each answer was calculated and graded as "good", "satisfactory", or "poor" and agreement over time of this "all raters grade" evaluated.

#### 2.2 Inter-rater agreement

To assess the inter-rater agreement within the group of four (for the content quality score) respectively five (for the formal quality score) raters, the percentage of expert answers all raters gave the same grade to, and the percentage of expert answers with one or two grades between the maximum and minimum grade given were calculated. To describe the bias of raters to systematically score lower or higher than the mean score, the percentage of expert answers which achieved more than one point, and more than two points above or below the mean score was calculated for each rater.

### 3. Evolution of quality of answers over time in the participating language zones

Two time periods were defined for analyzing potential trends of the quality of answers over time. The "early period" comprised the first ten expert answers from the beginning of the online period of the respective language zone. The "later period" comprised the last ten expert answers prior to the cut-off date (3^rd ^July 2009). The cut-off date was approximately two years after the ECORN-CF project was initiated in May, 2007 (see Table 1 for details of online dates, number of expert answers and time intervals). A reduced number of expert answers were included from two language zones due to a lower number of questions posted. The time span between the "early" and "later" periods varied from a minimum of approximately four months to a maximum of 18 months due to different dates of going online and different numbers of asked questions.

#### Statistical Analysis

Descriptive statistics (percentages) were used to summarize data. In order to describe intra- and inter-rater agreement, Cohen's weighted kappa values using linear weights and the respective standard errors were calculated according to Fleiss et al. [12] The kappa values were interpreted according to Landis and Koch (agreement: poor < 0.00; slight 0.00-0.20; fair 0.21-0.40; moderate 0.41-0.60; substantial 0.61-0.80; almost perfect 0.81-1.00) [13]. A p value of p < 0.05 was regarded to be significant.

To assess the bias of raters, the mean difference between the individual score given by a rater and the mean score of the group of raters was calculated. Two-sided t-test for paired data was used to determine statistically significant differences between individual scores and the mean score.

Two-sided t-tests were employed to compare mean scores for answers given during the early period to those given during the later period for all answers combined and for each language zone separately.

#### Ethics committee

No formal ethical approval was obtained as the study was not a matter of research on humans. All analyses were restricted to existing data from a data base.

## Results

### 1. Development of a quality assessment tool to analyze quality of expert answers

The quality assessment tool, scoring system and training manual are detailed in Table 2, and Additional File 1. The aspect "correctness of the content" was regarded to be of special importance. Therefore, this aspect was labeled with zero points for a "poor" answer, three for a "satisfactory" and six for a "good" answer, whereas in contrast, the other two aspects of content quality (completeness and openness) were scored with zero, one and two points for a "poor", "satisfactory" or "good" answer, respectively. If the content of the answer was not correct and achieved only zero points, the overall content quality of the respective answer was graded as "poor".

**Table 2:** Scoring system judging content and formal quality of expert answers

I. Content quality	Grade	Score (points)
I.1 Content correct, according to guidelines	Poor	0
	Satisfactory	3
	Good	6

I.2 Completeness of the answer, suitability	Poor	0
	Satisfactory	1
	Good	2

I.3 Openness (are rigid statements avoided without room for differing strategies)	Poor	0
	Satisfactory	1
	Good	2

**Total Score for content quality**	**Poor**	**0-3**
	**Satisfactory**	**4-7**
	**Good**	**8-10**

**II. Formal quality**	Grade	Score (points)

II.1 Comprehensive Style	Poor	0
	Satisfactory	1
	Good	2

II.2 Personal Style	Poor	0
	Satisfactory	1
	Good	2

II.3 Extent of answer	Poor	0
	Satisfactory	1
	Good	2

**Total Score for formal quality**	**Poor**	**0-2**
	**Satisfactory**	**3-4**
	**Good**	**5-6**

If I.1 "correctness of the content" was scored zero the answer was graded as poor overall.

### 2. Assessment of intra- and inter-rater agreement

#### 2.1 Intra-rater agreement

Concerning the content quality of answers, the intra-rater agreement differed widely between the individual raters. Results are shown in Table 3. The percentage of expert answers which were graded twice in complete congruence varied from 48-76% among raters, while the percentage of expert answers which showed a discrepancy of one grade ranged from 16-48% and two grades ranged from 0-5% among raters when scored twice. Rater 1 showed a only a slight, rater 4 a fair and raters 2 and 3 a moderate intra-individual agreement over time, estimated by weighted kappa values. Only for raters 2 and 3, a significant agreement over time was shown. When mean scores of all raters were analyzed 84% of expert answers had the same grade and the remaining had a difference of one grade when scored twice. In contrast to the individual scores, the grades based on group mean scores between two time points showed substantial agreement estimated by weighted kappa values (p < 0.001).

**Table 3:** Intra-rater agreement between two assessments over time of individual raters and of all raters pooled

Content quality				
	**Complete congruence**	**Discrepancy 1 grade**	**Discrepancy 2 grades**	**weighted kappa ± standard error**

**Rater 1**	11/23	11/23	1/23	0.020 ± 0.172
	(48%)	(48%)	(4%)	p = 0.909

**Rater 2**	19/25	6/25	0/25	0.559 ± 0.130
	(76%)	(24%)	(0%)	p < 0.001

**Rater 3**	17/25	8/25	0/25	0.460 ± 0.123
	(68%)	(32%)	(0%)	p < 0.001

**Rater 4**	13/22	8/22	1/22	0.236 ± 0.152
	(59%)	(36%)	(5%)	p = 0.120

**Mean all raters**	21/25	4/25	0/25	0.669 ± 0.149
	(84%)	(16%)	(0%)	p < 0.001

**Formal quality**				

	Complete congruence	Discrepancy 1 grade	Discrepancy 2 grades	weighted kappa ± standard error

**Rater 1**	10/25	12/25	3/25	0.145 ± 0.150
	(40%)	(48%)	(12%)	p = 0.336

**Rater 2**	20/25	4/25	1/25	0.650 ± 0.133
	(80%)	(16%)	(4%)	p < 0.001

**Rater 3**	13/25	11/25	1/25	0.147 ± 0.161
	(52%)	(44%)	(4%)	p = 0.360

**Rater 4**	11/22	8/22	3/22	0.000 ± 0.000
	(50%)	(36%)	(14%)	p = 1

**Rater 5**	19/24	4/24	1/24	0.410 ± 0.249
	(79%)	(17%)	(4%)	p = 0.100

**Mean all raters**	13/25	11/25	1/25	0.169 ± 0.150
	(52%)	(44%)	(4%)	p = 0.260

25 expert answers were scored at two different points in time by the same raters. The numbers represent the number of expert answers (percentage in brackets) that were scored with the same grade twice (complete congruence), that were scored the second time one grade lower or higher than the first time (discrepancy 1 grade) and that were scored two grades lower or higher the second time (discrepancy 2 grades). If the total number of expert answers is lower than 25, the respective rater regarded some of the expert answers as "unscorable". Rater 5 as a representative of the German CF-patient organization and not a care team member scored only the formal aspect of answers. The kappa values for agreement were interpreted according to the scale by Landis and Koch [13]: agreement poor < 0; slight 0.00-0.20; fair 0.21-0.40; moderate 0.41-0.60; substantial 0.61-0.80; almost perfect 0.81-1.00). A p value of p < 0.05 was regarded to be significant.

For scoring of the formal quality of answers, there was even less agreement between time points. The percentage of expert answers scored twice by an individual in complete congruence ranged from 40-80%, those scored with one grade difference ranged from 16-48% and those scored with two grades difference ranged from 4-14% among raters (Table 3). The kappa values revealed a slight agreement for raters 1,3 and 4, a moderate agreement for rater 5 and a substantial agreement for rater 2. Only for rater 2, a kappa value significantly different from zero was observed. When mean scores of all raters were analyzed, 52% of answers had the same grade and 44% and 4% had a difference of one and two grades respectively, when scored twice. The grades based on mean scores for formal quality showed only slight, non significant agreement between two time points.

### 2.2 Inter-rater agreement

Raters agreed on the same grade in 42% of the expert answers for content quality and in 26% for formal quality. There was a discrepancy of one grade between the highest and lowest grade given from one or more raters (e.g. "good" was the highest grade and "satisfactory" was the lowest grade given for the respective answer) in 50% of expert answers for content quality and in 38% of expert answers for formal quality. For content quality, 8% of expert answers got the highest grade "good" and the lowest grade "poor" form different raters. For formal quality, this was the case in 36% of expert answers. The inter-rater agreement was calculated from 106 out of the 108 expert answers with a complete set of scores from all raters available. For the content quality, the inter-rater agreement was fair (mean kappa value 0.232 ± 0.036, p < 0.001) while only slight agreement was observed for the grades of the formal quality (mean kappa value 0.105 ± 0.024, p < 0.001).

As individual raters judged a certain answer differently, we assessed the bias of individual raters to systematically score higher or lower than the mean score of the group. The results are shown in Table 4. In summary, the tendency of a single rater to score lower or higher than the mean score was consistent for content and formal quality. Two raters had a clear tendency to score below (differences between individual scores and the mean score were significant, except for the content score of one rater) and two raters a clear tendency to score above the mean with a significant difference to the mean score of the group. One rater was relatively in line with the mean of the group.

**Table 4:** Tendency of the individual raters to score above or below the mean score

Content quality						
	**% of answers 2 or more points below mean score**	**% of answers > 1 and < 2 points below mean score**	**% of answers > 1 and < 2 points above mean score**	**% of answers 2 or more points above mean score**	**Bias (score-mean score) (mean ± SD)**	**P (comparison score with mean score)**

**Rater 1**	17.6	7.4	8.3	5.6	-0.45 ± 1.53	0.003

**Rater 2**	12	7.4	10.2	7.4	0.03 ± 1.43	0.839

**Rater 3**	15.9	8.4	6.5	5.6	-0.25 ± 1.50	0.084

**Rater 4**	4.6	2.8	10.2	21.3	0.68 ± 1.52	< 0.001

**Formal quality**						

	% of answers 2 or more points **below **mean score	% of answers > 1 and < 2 points **below **mean score	% of answers > 1 and < 2 points **above **mean score	% of answers 2 or more points **above **mean score	Bias (score-mean score) (mean ± SD)	P (comparison score with mean score)

**Rater 1**	24.1	17.6	0.9	0	-0.97 ± 1.13	< 0.001

**Rater 2**	2.8	3.7	2.8	0.9	0.00 ± 0.71	1.000

**Rater 3**	9.3	9.3	4.7	0.9	-0.25 ± 0.98	0.012

**Rater 4**	1.9	0	22.2	6.5	0.66 ± 0.89	< 0.001

**Rater 5**	2.8	2.8	19.4	6.5	0.56 ± 0.94	< 0.001

The numbers represent the percentage of expert answers that were scored more than one respectively two points above/below the mean score from all raters. Rater 5 as a representative of the German CF-patient organization and not a care team member scored only the formal aspect of the expert answers. To assess the bias of raters, the mean difference between the individual score given by a rater and the mean score of the group of raters was calculated. A p value of p < 0.05 was regarded to determine statistically significant differences between individual scores and the mean score.

### 3. Evolution of quality of answers over time in the participating language zones

**Figure 1:**
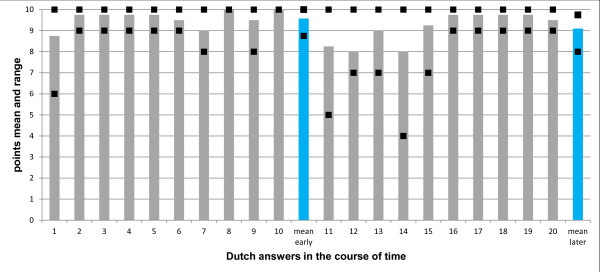
**Content quality of answers from the Dutch language zone during the early and later period of the ECORN-CF system**. Ten expert answers from the early period (1-10) and ten expert answers from the later period (11-20) were assessed. Each column represents the mean of the four scores given to a single answer (one additional rater scored only the formal quality). The squares represent the highest and lowest scores given to that answer. The blue columns at the end of each group of ten expert answers represent the mean of the scores for all answers of the early period and the later period, respectively; they did not differ between early and later period (p = 0.095). Dark grey columns represent answers with a mean score of "good" quality.

**Figure 2:**
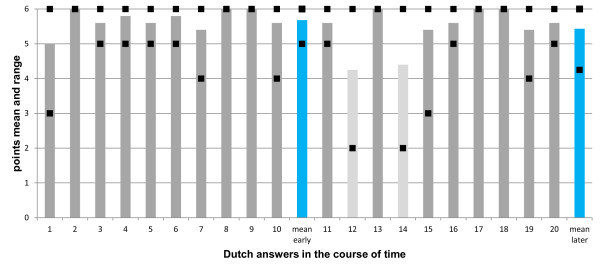
**Formal quality of answers from the Dutch language zone during the early and later period of the ECORN-CF system**. Ten expert answers from the early period (1-10) and ten expert answers from the later period (11-20) were assessed. Each column represents the mean of the five scores given to a single answer. The squares represent the highest and lowest scores given to that answer. The blue columns at the end of each group of ten expert answers represent the mean of the scores for all answers of the early period and the later period, respectively; they did not differ between early and later period (p = 0.265). Dark grey columns represent answers with a mean score of "good" quality, light grey columns those of "satisfactory" quality.

Figures 1 and 2 show as an example the content and formal quality for the Dutch language zone (for questions from Belgium and the Netherlands). The mean score for content quality decreased slightly from the early to the later period (from 9.6 to 9.1 points), however the overall content quality of expert answers from both periods clearly were on a "good" level and did not differ statistically significant over time (p = 0.095, Figure 1). Concerning the formal quality, all expert answers from the early period were scored as "good", and only two of ten from the later period were scored as "satisfactory" (Figure 2). The mean score for formal quality also decreased slightly from the early to the later period (5.7 vs. 5.4 points, respectively), however the grades based on those mean scores from the two periods clearly were on a "good" level and did not differ over time (p = 0.265).

**Figure 3:**
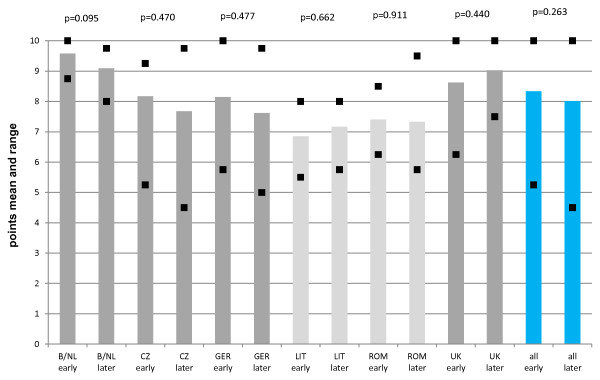
**Content quality of answers in all six language zones during the early and later period of the ECORN-CF system**. Each column represents the mean of all scores for the expert answers from the respective period marked as "early" or "later" and the respective language zone. The squares represent the highest and lowest mean score given to a single answer. The blue columns marked as "all" represent the mean of the scores for all 50 expert answers from all language zones of the early period and for all 58 expert answers from all language zones of the later period, respectively. Dark grey columns represent answers of "good" quality, light grey columns those of "satisfactory" quality. B/NL = Belgium/Netherlands, CZ = Czech Republic, GER = Germany, LIT = Lithuania, ROM = Romania, UK = United Kingdom.

**Figure 4:**
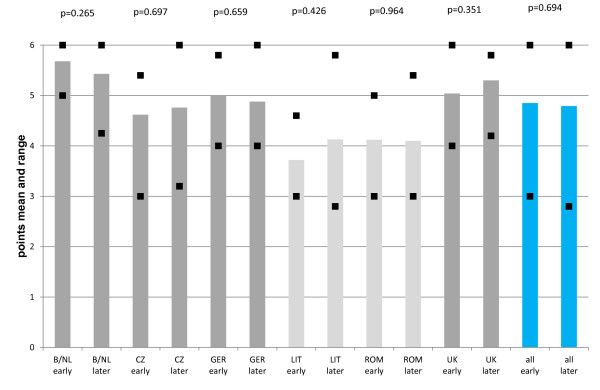
**Formal quality of answers in all six language zones during the early and later period of the ECORN-CF system**. Each column represents the mean of all scores for the expert answers from the respective period marked as "early" or "later" and the respective language zone. The squares represent the highest and lowest mean score given to a single answer. The blue columns marked as "all" represent the mean of the scores for all 50 expert answers from all language zones of the early period and for all 58 expert answers from all language zones of the later period, respectively. The squares represent the highest and lowest mean score given to a single answer. Dark grey columns represent answers of "good" quality, light grey columns those of "satisfactory" quality. B/NL = Belgium/Netherlands, CZ = Czech Republic, GER = Germany, LIT = Lithuania, ROM = Romania, UK = United Kingdom.

A summary of the content/formal quality of answers from all participating language zones at the early and later period is shown in Figures 3 and 4. The Dutch and English language zones were leading in respect of the content quality of answers (Figure 3), with "good" quality level during both periods. The Czech and German language zone showed-on average-a bearly "good" content quality and the Romanian and Lithuanian language zone a "satisfactory" content quality in both periods. The formal quality of answers (Figure 4) follows a similar pattern with a clear "good" quality for the Dutch and English answers, a "good" and bearly "good" quality for the German and Czech answers, and a "satisfactory" quality for the Lithuanian and Romanian answers. Taking all language zones together, the content quality of answers remained on a "good" quality level (Figure 3) and the formal quality on a bearly "good" level (Figure 4) during the early and later period. There were no significant differences from the early to the later period in any of the language zones or in the group of language zones taken together.

## Discussion

This study describes the development and use of a quality assessment tool for expert answers provided on an Internet platform as part of the ECORN-CF project. This tool assesses the content and formal quality of answers, with a training manual available to guide raters. The intra-rater agreement for both, content and formal quality when scoring the same answer twice, one year apart, showed poor agreement however, when group mean scores of an expert panel were used there was substantial agreement over time for content quality but not for formal quality. Within the group of raters, the inter-rater agreement for scoring the content quality was fair, whilst it was only slight concerning the score for formal quality. Furthermore, some raters showed a substantial bias towards high or low scores. Therefore, it becomes clear that in order to overcome the intra- and inter-rater variability a group of raters is needed. However, the intra- and inter-rater agreement of scores for formal quality of an answer could not be improved by using the group mean score. Therefore, the quality assessment tool presented in this study seems most adequate for the judgment of content quality of expert answers.

There were some expert answers that scored "good" content quality at one time and then "poor" content quality at another time by the same person (two out of 25 expert answers) and there were also some that scored "good" content quality by some raters and "poor" by others (nine out of 108 expert answers). So in total eleven answers were scored highly discrepantly. A number of factors may have contributed to this. One explanation might be the lack of standards for a certain topic. At our quality round table meetings, discrepant answers were discussed with the experts/raters to identify reasons why the raters came to such differing results. After a detailed analysis of topics of those answers, it became evident that most with divergent scores dealt with topics where no standards or guidelines were available (eight out of eleven answers). In these situations expert advice is likely to be influenced by "access to best evidence", local practice and personal bias. This emphasizes the need to establish clear consensus statements/guidelines to ensure local practice is evidence based and consistent across centres. ECORN-CF offers the opportunity to identify topics, for which there is an urgent need to develop unanimous recommendations. This identification process mainly takes part during the control process of all answered questions in the English archive as described in the Background section. One concrete result of this process is the recently published European paper on "Travelling with cystic fibrosis" [14]. Other projects will follow leading to more unequivocal counseling and treatment strategies for patients with CF all over Europe.

Other factors which may have contributed to poor agreement between individuals and over time include the raters' professional background (pediatricians, physiotherapist, representative of CF-patient organization), country of origin, exposure to lay questions and expert answers over the time frame of the project. Training of the expert panel undertaking quality assessment (e.g. training seminars and teaching manuals) is key to ensure a valid estimate of quality of expert advice. Another goal which we pursued with the implementation of this quality assessment tool was to gather information about the quality of answers in each language zone and its development over time. We did not show an improvement of quality of answers over the time frame of the study and this is likely attributed to the short time frame between the early and later periods as well as the low numbers of questions in some language zones. Furthermore, four out of six language zones already had a "good" quality level of answers during the early period, which remained "good" during the later period.

The quality assessment of expert answers revealed that, when taking all answers from all language zones, the overall content and formal quality was on a good level in both periods. However, in two out of six participating language zones (i.e. the Eastern European Member States Romania and Lithuania), the level of quality of answers remained on a "satisfactory" level. Unlike some countries Romania and Lithuania had no experience with such a platform until the start of the ECORN-CF project, the number of questions asked on those platforms was quite low compared to other language zones and the time between the early and later period was relatively short, so that the possibility to gain experience in order to improve the quality of answers was lacking.

It seems to be of great importance to continue the ECORN-CF project, in order to really achieve the anticipated aim: facilitating access to specific information for patients with CF in all member state languages at the same highest level of quality.

The best scores for content and formal aspects of answers were achieved in the Dutch language zone which also did not have prior experience with Internet patient advice platforms. The Dutch language zone had a unique approach to development of expert answers. A local team of medical residents/registrars were encouraged to develop an answer which was then discussed in a group forum. A standardized format was used for group discussions which included: what aspects are desirable to appear in the answer?; how is the given answer judged from the other members of the group?; is the content correct, what is lacking? All this information was used to develop a complete answer which was approved by the moderator before submission to the ECORN-CF platform. Adoption of similar processes in other language zones would improve quality and consistency of answers in the ECORN-CF project.

Training on how best to provide expert advice is a core part of a successful Internet patient advice platform. Many ways appeared to be possible: training supported by short term visits e.g. according to the "Pendleton Rules". Using these rules the expert answering the question discusses what he did well, then the trainer discusses what the expert did well, before he is allowed to become critical. The expert describes what could have been done differently and makes suggestions for change. Another possibility could be the initiation of workshops focusing on how to give answers of good quality. Furthermore, it would be of great importance to involve the patient organizations, e.g. for getting help in recruiting the right experts for special topics or for combining quality training with conferences.

Many Internet platforms where patients are able to ask questions about specific illnesses are existing [8-11]. Current studies have focused on the reasons why patients turn to the Internet, how happy they are with the answers and what kind of information they seek. To our knowledge there are no studies on quality control within these platforms.

With increasing access in both EU and non-EU countries, the Internet is now an important patient platform for health care advice [6,7]. As a consequence, there is increased need for quality control of such Internet information platforms. The quality assessment tool in this study is suitable for content quality control of expert answers and a modified version could be used in other expert advice Internet platforms. A mechanism to recognize Internet platforms with rigorous inbuilt quality control mechanisms (e.g. through a kite marking system) would be useful so that patients could be confident that they are receiving high quality advice.

## Conclusions

This study describes the development and use of a quality assessment tool for expert answers to lay questions within the ECORN-CF Internet platform. This tool is suitable for the assessment of content quality of answers, as intra- and inter-rater variability could be leveled by using the pooled score out of a group of four raters.

The quality of expert answers was high in most language zones, with improvements possible in other language zones. Implementation of strategies to improve the quality of expert advice are important. The quality assurance mechanisms inbuilt into ECORN-CF will ensure that CF patients all over Europe have equal possibility of getting access to high quality expert advice on their illness.

## Competing interests

The authors declare that they have no competing interests.

## Authors' contributions

TOFW mainly designed the ECORN-CF project with AP coordinating it including the training of the experts by sample questions and answers, and organizing quality round tables. HH and DD were responsible for the work package "answers to lay questions" within the ECORN-CF project. DD and HH developed the first version of the scoring system which was adjusted and finalized with input from all co-authors. HH, KB, VV, JB and BD scored the expert answers. DD analyzed the data and drafted the manuscript. All authors critically reviewed the manuscript and read and approved the final version.

## Pre-publication history

The pre-publication history for this paper can be accessed here:

http://www.biomedcentral.com/1471-2288/12/11/prepub

## Supplementary Material

Additional file 1 **Training manual for quality assessment of expert answers according to the newly developed scoring system**. This guide gives a detailed description with examples how to assess the content and formal quality of an expert answer. The scoring system is introduced which comprises points given to each aspect of content and formal quality and the calculation of the final content and formal score is explained. Click here for file
