# Supplementary material for: Quality assessment of expert answers to lay questions about cystic fibrosis from various language zones in Europe: the ECORN-CF project
Source: BMC Med Res Methodol. 2012 Feb 6;12:11. doi: 10.1186/1471-2288-12-11 (PMC3311602; doi:10.1186/1471-2288-12-11)
Supplement: Additional file 1 — Training manual for quality assessment of expert answers according to the newly developed scoring system. This guide gives a detailed description with examples how to assess the content and formal quality of an expert answer. The scoring system is introduced which comprises points given to each aspect of content and formal quality and the calculation of the final content and formal score is explained. [file 1471-2288-12-11-S1.DOC]

# Quality assessment of expert answers to lay questions about cystic fibrosis from various language zones in the ECORN-CF project

Daniela d’Alquen^1§^, Kris De Boeck^2^, Judy Bradley^3^, Věra Vávrová^4^, Birgit Dembski^5^, Thomas OF Wagner^6^, Annette Pfalz^6^, Helge Hebestreit^1^

^1^Department of Pediatrics, University Hospital Wuerzburg, Josef-Schneider-Str. 2, 97080 Wuerzburg, Germany

^2^Pediatric Pulmonology, Department of Pediatrics, University Hospital of Leuven, Herestraat 49, 3000 Leuven, Belgium

^3^Health and Rehabilitation Sciences Research Institute, University of Ulster and Adult CF Centre BHSCT, Shore Road Newtownabbey, Northern Ireland BT37 0QB

^4^Cystic Fibrosis Centre, Charles University, 2^nd^ School of Medicine, V Uvalu 84, 15006 Prague 5, Czech Republic

^5^CF Europe, Bonn, and CF Association “Mukoviszidose e.V.”, In den Dauen 6, 53117 Bonn, Germany

^6^Department of Pneumology, Goethe University Hospital Frankfurt, Theodor-Stern-Kai 7, 60590 Frankfurt am Main, Germany

# - Additional file 1 -

# Training manual for quality assessment of expert answers according to the newly developed scoring system

I: Content quality

## I.1. Content correct/According to guidelines?

Poor: 0 points

Satisfactory: 3 points

Good: 6 points

**EXAMPLE:**

Q: How often do you advise I should see my CF doctor?

A1: Dear questioner, it would be sufficient if you see your CF doctor only when you have clinical symptoms / problems.

***🡪 Content not correct; 0 points***

A2: Dear questioner, according to European guidelines we would recommend that patients should see their CF doctor every 1-3 month, preferably every month. Newly diagnosed infants or patients with severe disease should be seen more often, and those with mild disease or atypical CF may be seen less often, every 3-6 months.

***🡪 Content correct, according to guidelines, 6 points***

## I.2. Completeness of answer/ Suitability

Does the text answer the question at all? Are all aspects of the question covered?

Poor: 0 points

Satisfactory: 1 point

Good: 2 points

**EXAMPLE:**

Q: My 7-year-old son suffers from CF. We saw the ENT-specialist who told us that the hearing test was pathologic. He explained that this might be caused by hypertrophic adenoids or by mucus accumulating in the middle-ear, not able to flow off. He mentioned an operation of the adenoids or the tympanic membrane and we should see him again in 3 weeks. I asked the doctor if we should try to do inhalations with camomile within the next 3 weeks, but he did not really give an answer. I would like to know if this accumulation of mucus in the middle ear is typical for CF and what is done with the operation, respectively what we can do to avoid an operation.

***🡪 So we have three aspects of the question:***

1. ***Is the accumulation of mucus in the middle ear typical for CF?***
2. ***What is done with the operation?***
3. ***What can we do to avoid the operation?***

A: Dear questioner,

An accumulation of fluid behind the tympanic membrane is a common problem in childhood. **According to all investigations done so far it is not more frequent in children with CF than in the general population (1.).** The accumulation of fluid is caused by swelling of the tube connecting the middle ear cavity and the throat. This swelling results in absorption of the air in the middle ear and replacement by fluid.

The therapy of the accumulation of fluid behind the tympanic membrane contains a clinical control after (1-)3 months, because the effusion disappears very often without any therapy. If there are any complications in the meantime (e.g. otitis media or delay in speech development) or if the fluid persists, **a cut in the tympanic membrane is done (paracentesis). If there is repeated accumulation of fluid, one has to think about putting a tube into the tympanic membrane or abolish the underlying problems, e.g. perform an operation of adenoids (2.).**

A drug therapy of the fluid in the middle ear can not be recommended, as studies could not show any therapeutic effects. Similarly there is no proof of evidence for the inhalation of camomile. Therefore, we would not recommend such a therapy to our patients, especially since the possibility of allergic reactions can not be ruled out. If breathing through the nose is not possible, we would recommend constrictive nose drops (not spray), for reopening the connection between middle ear and throat, so that the fluid can flow off. But there is no scientific proof of efficacy for this kind of therapy, either(3.).

***🡪 All aspects of the question have been covered, 2 points***

## I.3. Openness

Does the answer avoid rigid statements regarding future therapy and words like “must“ or “has to”? Are words like “should“ or “could“ used instead?

Poor: 0 points

Satisfactory: 1 point

Good: 2 points

**EXAMPLE 1:**

Q: Dear ladies and gentlemen,

My little daughter Linea suffers from cystic fibrosis. Up to now we heard from all sides including the doctors that pseudomonas bacteria are especially dangerous. Because our daughter coughed a lot we did a swab culture from the throat on the 23^rd^ of March, 2007. The result showed among others a *Pseudomonas stuzeri*. The doctor in charge says, that these bacteria are not dangerous and, therefore, no therapy is required. I am sorry for not being able to believe the correctness of this information. Therefore I would like to ask for your help with this problem.

A1: Dear questioner, according to our long-term experience, the bacterium *Pseudomonas stutzeri* is rarely found in CF-patients. In contrast to *Pseudomonas aeruginosa*, there is no patient in whom this bacterium could be detected for a long period of time. Nevertheless we are not of the opinion of your doctor and think that this bacterium must be treated if there are any clinical symptoms.

This answer is not very open, there is no room for a compromise between the opinion of the local CF specialist and the opinion of the expert here; it is important to work together with the local CF-specialist and not against each other, so it is important to refer the patient to their local CF-centre. 0 points

A2: Dear questioner, according to our long-term experience, the bacterium *Pseudomonas stutzeri* is rarely found in CF-patients. In contrast to *Pseudomonas aeruginosa*, there is no patient in whom this bacterium could be detected for a long period of time. Nevertheless, we usually have treated the patients with effective antibiotics, if there were clinical signs of an infection, e.g. coughing.

There is no specific evidence in the scientific literature for a pathogenic role of *Pseudomonas stutzeri*. It is likely that your doctor therefore decided not to initiate any treatment. On the other hand, this bacterium is more often found in patients suffering from immunodeficiency. As a rule, these patients are treated. Probably you could contact your local CF-centre and could talk to your doctor again, whether a 14-day therapy with an oral antibiotic should be initiated, especially in view of the actual clinical symptoms***.***

*** Even if there is no clear right or wrong answer to this question, the answer is a compromise and helps not to lose confidence in the local CF-team. Please recommend as often as possible to contact the local CF-team. 2 points***

**EXAMPLE 2:**

Q: My daughter has a newly diagnosed CF and we are looking for a CF centre nearby and with good care for her. Where can we go to?

A: Dear questioner, please contact the local CF-patient organisation which usually provides a list of all CF-centres and much more other helpful information.

*** Please refer the patient as often as possible to the local patient organisation!***

**EXAMPLE 3:**

Q: What is the latest therapy regimen for young adults with CF and osteoporosis? Please give information about drugs and dosage.

A: Dear collegue, as this is a public forum we are not allowed to give the names of special drugs. We still recommend to adapt the therapy to the risk of fracture***….***

*** Please notify that we are not allowed to give the names of special drugs!!***

# II. Formal quality

## II.1. Comprehensive Style

Is the speech of the answer understandable for the patient/ layperson?

Poor: 0 points

Satisfactory: 1 points

Good: 2 points

### EXAMPLE:

Q: My 7-year-old son suffers from CF. We saw the ENT-specialist who told us that the hearing test was pathologic. He explained that this might be caused by hypertrophic adenoids or by mucus accumulating in the middle-ear, not able to flow off. He mentioned an operation of the adenoids or the tympanic membrane and we should see him again in 3 weeks. I asked the doctor if we should try to do inhalations with camomile within the next 3 weeks, but he did not really give an answer. I would like to know if this accumulation of mucus in the middle ear is typical for CF and what is done with the operation, respectively what we can do to avoid an operation.

A1: Dear questioner,

An accumulation of fluid behind the tympanic membrane is a common problem in childhood. It can be caused by swelling of the Eustachian tube due to hypertrophic adenoids. According to all investigations done so far it is not more frequent in children with CF than in the general population.

The therapy of the accumulation of fluid behind the tympanic membrane includes a clinical control after (1-)3 months. If the fluid persists, a paracentesis is done. If there is repeated accumulation of fluid, one has to think about abolishing the underlying problems, e.g. perform an adenotomy.

***🡪 Explanation of the anatomy or the operation technique not understandable for lay persons, too many technical terms are used: e.g. Eustachian tube, hypertrophic adenoids, paracentesis, adenotomy, 🡪 0 points***

A2: An accumulation of fluid behind the tympanic membrane is a common problem in childhood. According to all investigations done so far it is not more frequent in children with CF than in the general population. The accumulation of fluid is caused by swelling of the tube connecting the middle ear cavity and the throat. This swelling results in absorption of the air in the middle ear and replacement by fluid.

The therapy of the accumulation of fluid behind the tympanic membrane includes a clinical control after (1-)3 months, because the effusion disappears very often without any therapy. If there are any complications in the meantime (e.g. otitis media or delay in speech development) or if the fluid persists, a cut in the tympanic membrane is done (paracentesis). If there is repeated accumulation of fluid, one has to think about putting a tube into the tympanic membrane or abolish the underlying problems, e.g. perform an operation of adenoids.

***🡪 Here understandable explanation without too many technical terms, 2 points***

## II.2. Personal Style

Is the answer written in a personal, friendly way addressing the patient?

Poor: 0 points

Satisfactory: 1 points

Good: 2 points

***🡪 Address to the patient in a friendly way; but be aware that you should not use the personal name of the patient. Even if he has signed the e-mail by e.g. “Mr. Miller”, do not address: “Dear Mr. Miller”.(Even if he has signed his e-mail, he should not want after his question being published on a website, that everybody can read his name).***

For example use instead: ”Dear questioner”.

**EXAMPLE:**

Q: How often do I have to present my son at the local CF-centre?

A1: Present at local CF-centre 4 times a year.

***🡪 Not personal, telegram style, 0 points***

A2: Dear questioner, we would recommend that you present your son at the local CF-centre 4 times a year. We hope that we were able to help you. Yours sincerely, …

***🡪 Here the questioner is addressed friendly; mentioning “the son” directly gives the answer a personal style; some nice phrases for ending the answer.🡪 2 points***

## II.3. Extent of answer

Is the extent of the answer suitable for the question? Is it detailed enough?

Poor: 0 points

Satisfactory: 1 points

Good: 2 points

### EXAMPLE:

Q: My 7-year-old son suffers from CF. We saw the ENT-specialist who told us that the hearing test was pathologic. He explained that this might be caused by hypertrophic adenoids or by mucus accumulating in the middle-ear, not able to flow off. He mentioned an operation of the adenoids or the tympanic membrane and we should see him again in 3 weeks. I asked the doctor if we should try to do inhalations with camomile within the next 3 weeks, but he did not really give an answer. I would like to know if this accumulation of mucus in the middle ear is typical for CF and what is done with the operation, respectively what we can do to avoid an operation.

A1: Dear questioner, accumulation of fluid behind the tympanic membrane is not typical for CF. If this is due to hypertrophic adenoids, the adenoids have to be taken away by an operation. There is no real evidence based conservative therapy; if the fluid does not flow off by itself within several weeks, an operation is recommended.

***🡪 Answer covers all aspects of the question, but very shortly, no explanations are given. 0 points***

A2: Dear questioner,

An accumulation of fluid behind the tympanic membrane is a common problem in childhood. According to all investigations done so far it is not more frequent in children with CF than in the general population. The accumulation of fluid is caused by swelling of the tube connecting the middle ear cavity and the throat. This swelling results in absorption of the air in the middle ear and replacement by fluid.

The therapy of the accumulation of fluid behind the tympanic membrane contains a clinical control after (1-)3 months, because the effusion disappears very often without any therapy. If there are any complications in the meantime (e.g. otitis media or delay in speech development) or if the fluid persists, a cut in the tympanic membrane is done (paracentesis). If there is repeated accumulation of fluid, one has to think about putting a tube into the tympanic membrane or abolish the underlying problems, e.g. perform an operation of adenoids.

A drug therapy of the fluid in the middle ear can not be recommended, as studies could not show any therapeutic effects. Similarly there is no proof of evidence for the inhalation of camomile. Therefore, we would not recommend such a therapy to our patients, especially since the possibility of allergic reactions can not be ruled out. If breathing through the nose is not possible, we would recommend constrictive nose drops (not spray), for reopening the connection between middle ear and throat, so that the fluid can flow off. But there is no scientific proof of efficacy for this kind of therapy either.

🡪 Here the extent is suitable for the answer, with a lot of information and explanation, 2 points

**Total Score for content quality:**

I.1. Content correct/According to guidelines?: max. 6 points

I.2. Completeness of answer/ Suitability: max. 2 points

I.3. Openness: max. 2 points

## Max. total score: 10 points

**0-3 points: poor**

**4-7 points: satisfactory**

**8-10 points: good- very good**

(If I.1. =0 points, the complete score for content quality is poor)

**Total Score for formal quality:**

II.1. Comprehensive Style: max. 2 points

II.2. Personal Style: max. 2 points

II.3. Extent of answer: max. 2 points

## Max.total score: 6 points

**0-2 points: poor**

**3-4 points: satisfactory**

**5-6 points: good- very good**
